# Efficacy and Safety of Perioperative Transcranial Electrical Stimulation for Postoperative Pain and Related Outcomes: A Systematic Review and Meta‐Analysis With Trial Sequential and Subgroup Analyses

**DOI:** 10.1155/prm/4293899

**Published:** 2026-10-23

**Authors:** Zehao Liu, Yixin Zhang, Yangyang Li, Dezhou Jiang, Funing Liu, Chunmei Wu, Xiaolan Li, Suying Li, Wenjie Mao, Qing Zhong

**Affiliations:** ^1^ Department of Anesthesiology The Affiliated Hospital Southwest Medical University Luzhou Sichuan 646000 China swmu.edu.cn; ^2^ Department of Anesthesiology and Pain Medicine The People’s Hospital of Jianyang City Jianyang Sichuan 641400 China jy120.cn; ^3^ Graduate School Chengdu Medical College Chengdu Sichuan China cmc.edu.cn

**Keywords:** meta-analysis, neuromodulation, perioperative period, postoperative pain, systematic review, transcranial electrical stimulation

## Abstract

**Objectives:**

Transcranial electrical stimulation (tES) is a neuromodulation technique that can serve as an adjuvant tool during the perioperative period. With emerging randomized controlled trials (RCTs) supporting its efficacy across various surgical procedures, synthesis of current evidence and evaluation of its potential for postoperative analgesia in elective‐surgery patients and related outcomes are urgently needed.

**Materials and Methods:**

We initially searched PubMed, the Cochrane Library, MEDLINE, and Embase for RCTs investigating perioperative tES in patients undergoing elective surgery. The search period covered from the inception of each database to September 2025, and an updated search for newly published studies was conducted in September 2026. We analyzed the outcomes, including postoperative pain scores, opioid consumption, quality of recovery, incidence of postoperative delirium, and adverse events. The Cochrane Risk of Bias 2 (RoB 2) tool was used to evaluate the risk of bias in included studies. Sensitivity and publication bias analyses were performed. Exploratory subgroup analysis and meta‐regression were performed to identify sources of heterogeneity. We also analyzed outlier studies. Trial sequential analysis (TSA) was further performed to evaluate the robustness of evidence. The Grading of Recommendations Assessment, Development, and Evaluation (GRADE) system was used to assess the certainty of the evidence.

**Results:**

A total of 21 RCTs with 2186 participants were included in this meta‐analysis. Compared with the control group, the tES group showed lower pain scores on postoperative day 1 (standardized mean difference [SMD] = −0.57, 95% CI: −0.84 to −0.30, *p* < 0.01), reduced total opioid consumption (SMD = −0.47, 95% CI: −0.74 to −0.20, *p* < 0.01), a lower incidence of postoperative delirium (risk ratio [RR] = 0.45, 95% CI: 0.25 to 0.79, *p* < 0.01), and a lower incidence of postoperative nausea or vomiting (RR = 0.84, 95% CI: 0.75 to 0.95, *p* < 0.01). No significant differences were observed between the two groups in postoperative day 1 quality of recovery (SMD = 0.44, 95% CI: −0.18 to 1.05, *p* = 0.16) and dizziness or headache (RR = 1.01, 95% CI: 0.70 to 1.47, *p* = 0.94). In addition, tES might increase the risk of mild and transient stimulation site discomfort (RR = 3.10, 95% CI: 1.96 to 4.90, *p* < 0.01). The initial timing and number of stimulation sessions were potential sources of heterogeneity for postoperative day 1 pain scores, while initial stimulation timing, stimulation modality, and target sites were associated with heterogeneity in total opioid consumption. TSA results indicated that the current sample size was sufficient to confirm the beneficial effects of tES on postoperative day 1 pain, total opioid consumption, and postoperative delirium. The certainty of evidence ranged from very low to moderate.

**Conclusions:**

Current evidence suggests that tES, as an adjuvant perioperative intervention, may alleviate postoperative pain, reduce opioid consumption, and decrease the incidence of postoperative delirium. However, the effect of tES on postoperative quality of recovery remains unclear. Considering the limited overall quality of current evidence, whether tES can serve as a universal adjuvant analgesic method for patients undergoing elective surgery still needs further clarification.

## 1. Introduction

With population aging and the increasing burden of chronic diseases, the global surgical volume has significantly increased. A previous study reported that the global surgical volume exceeded 310 million procedures in 2012 and is expected to have increased substantially by 2030 with improved access to healthcare [1]. Pain is one of the most common postoperative complications. It has been reported that 48.7% of patients experience moderate to severe pain on the first postoperative day, and a considerable proportion have inadequate pain control [2]. Poor pain control increases opioid consumption, which increases the risk of respiratory depression, nausea, vomiting, addiction, and dependence. It may also prolong hospital stay, increase medical costs, and delay overall recovery [3, 4]. With the development of multimodal analgesia and enhanced postoperative recovery, traditional single‐drug analgesia strategies are no longer sufficient. Therefore, safer and more effective methods for postoperative pain management are required [5, 6].

In recent years, noninvasive brain stimulation techniques such as transcranial electrical stimulation (tES) have received increasing attention for perioperative pain management. tES modulates brain function by applying electrical currents through electrodes placed on the scalp [7]. Due to its noninvasive, safe, and cost‐effective characteristics, it has gradually been applied in the field of perioperative analgesia [8]. Several randomized controlled trials (RCTs) have demonstrated that tES can significantly reduce postoperative pain scores, decrease opioid consumption, and modulate brain electrical activity to reduce the incidence of postoperative delirium and accelerate postoperative recovery [9–12].

A recent meta‐analysis has evaluated the effects of transcranial direct current stimulation (tDCS) in patients undergoing general anesthesia [13]. Another meta‐analysis specifically assessed the efficacy of tDCS for arthroplasty [14]. However, there is currently no conclusive evidence regarding whether factors including different tES modalities, surgical types, and anesthesia types are associated with the efficacy of perioperative tES application. Therefore, this study aims to evaluate the efficacy and safety of tES for postoperative pain management in elective surgery, as well as its impacts on opioid consumption, quality of recovery, postoperative delirium, and the incidence of postoperative complications.

## 2. Methods

This review was registered with PROSPERO (CRD420261298593) [15]. The paper was prepared following the Preferred Reporting Items for Systematic Reviews and Meta‐Analyses (PRISMA) guidelines [16]. The certainty of evidence was assessed using the Grading of Recommendations Assessment, Development, and Evaluation (GRADE) approach [17].

### 2.1. Literature Search

We searched PubMed, Cochrane Library, MEDLINE, and Embase from database inception to September 2025, with an updated search for newly published studies performed during the manuscript revision process (September 2026). The search terms included “transcranial electrical stimulation,” “postoperative period,” “random allocation,” and related variants. The detailed search strategies for each database are provided in Supporting Table 1.

### 2.2. Inclusion and Exclusion Criteria

Studies satisfying the following criteria were included: (1) participants: patients undergoing elective surgery, with no restrictions on the type of surgery or anesthesia; (2) intervention: active‐tES; (3) comparison: sham‐tES; (4) outcomes: postoperative pain scores, postoperative opioid consumption, quality of recovery, incidence of postoperative delirium, and adverse events (nausea or vomiting, dizziness or headache, and stimulation site discomfort); and (5) study design: RCTs.

Studies were excluded for the following reasons: (1) duplication; (2) unreasonable study design; (3) full text unavailable; (4) completely missing data; (5) publication in a language other than English.

### 2.3. Study Screening and Data Extraction

Two reviewers (Y.Z. and Y.L.) independently screened the studies based on the predefined inclusion and exclusion criteria. Any disagreements were resolved through discussion with a third reviewer (S.L.). Two reviewers (D.J. and F.L.) independently extracted data using a standardized form. Disagreements were resolved by discussion with a third reviewer (W.M.). The extracted data included author names, year, country, type of surgery, primary anesthesia method, sample size, age, intervention details, and outcomes. Given the wide application of the Visual Analogue Scale (VAS) and Numeric Rating Scale (NRS) and their high correlation, both scales were included in the analysis of postoperative pain scores [18]. Since resting pain is the most basic pain outcome and has relatively consistent assessment standards, we selected resting pain scores for analysis when studies reported pain under different conditions (rest, movement, or coughing) [19]. One study [9] reported pain scores measured in both the morning and afternoon; we chose the afternoon pain scores to obtain more conservative pooled results (Supporting Table 2).

### 2.4. Risk of Bias Assessment

Two reviewers (C.W. and X.L.) used the Cochrane Risk of Bias 2 (RoB 2) tool to evaluate the risk of bias for each study across outcomes [20]. Disagreements were resolved by a third reviewer (Q.Z.). RoB 2 assesses bias in five domains: randomization process, deviations from intended interventions, missing outcome data, outcome measurement, and selection of reported results. Risk of bias summary figures were generated using the robvis tool [21].

### 2.5. Data Analysis

We used a graph reading tool (GetData Graph Digitizer 2.26) to estimate the data when the outcome data were reported graphically. We also applied the conversion formulas proposed by Wan and Luo et al. to estimate the mean and standard deviation (SD) when data were reported as medians with interquartile ranges (IQR) or ranges (minimum–maximum) [22, 23]. Pain scores were standardized to a scale of 0–10. Postoperative opioid consumption was converted to intravenous morphine milligram equivalents (MMEs), where applicable [24]. The primary outcome was the pain score on the first postoperative day.

Meta‐analyses were performed using the RevMan 5.4 software, Stata 18.0, and R 4.4.2. Continuous outcomes were pooled using the inverse variance method, and dichotomous outcomes were pooled using the Mantel–Haenszel method. The standardized mean difference (SMD) was used for continuous outcomes, and risk ratio (RR) was used for dichotomous outcomes. The results are presented as 95% confidence intervals (CIs). A random‐effects model was applied to all outcomes. Heterogeneity was assessed using Higgins *I* ^2^ and *Q* tests. *I* ^2^ values of ≥ 50% or *p* ≤ 0.1 indicated considerable heterogeneity. To explore the sources of heterogeneity, we performed post hoc subgroup analyses according to surgery type (orthopedic or nonorthopedic), anesthesia type (general anesthesia or regional anesthesia), initial timing of stimulation (preoperative, intraoperative, or postoperative), stimulation modality (transcranial alternating current stimulation [tACS] or tDCS), stimulation target sites (primary motor cortex [M1] and dorsolateral prefrontal cortex [DLPFC]), stimulation intensity (< 2 or 2 mA), and use of patient‐controlled analgesia (PCA). Meta‐regression was used to examine these factors as well as the number of stimulation sessions. Baujat plots and Graphical display of study heterogeneity (GOSH) plots were performed to detect potential outlier and influential studies [25]. Sensitivity analyses were conducted by excluding individual studies to assess their impact on the overall conclusions. Egger’s test was used to assess publication bias when the number of included studies for an outcome was sufficient. For outcomes with a small number of included studies (*n* < 10), funnel plots alone were applied for a preliminary evaluation of study symmetry. *p* < 0.05 denoted statistical significance.

Outcome certainty was assessed using the GRADE framework [17]. We performed the analysis with the GRADEpro Guideline Development Tool (GDT) (McMaster University, Ontario, Canada).

Trial sequential analysis (TSA) was performed to evaluate the robustness of evidence. The analysis was conducted using TSA software (version 0.9.5.10 beta). A random‐effects model with the DerSimonian–Laird method was applied. The monitoring boundaries were established based on the O’Brien‐Fleming α‐spending function with a two‐sided alpha of 0.05 and a statistical power of 80%.

## 3. Results

### 3.1. Literature Search

Initially, 500 studies were retrieved from the four electronic databases. After excluding 194 duplicate records, the titles and abstracts of the remaining articles were screened, and 284 irrelevant studies were further excluded. The full texts of the remaining 22 records were assessed for eligibility based on the predefined inclusion and exclusion criteria, of which 15 studies were eligible. Additionally, six eligible studies were identified through updated literature search during the manuscript revision phase. Ultimately, 21 studies were included in the final quantitative analysis. The literature screening flowchart and detailed exclusion reasons are presented in Figure 1.

**FIGURE 1:**
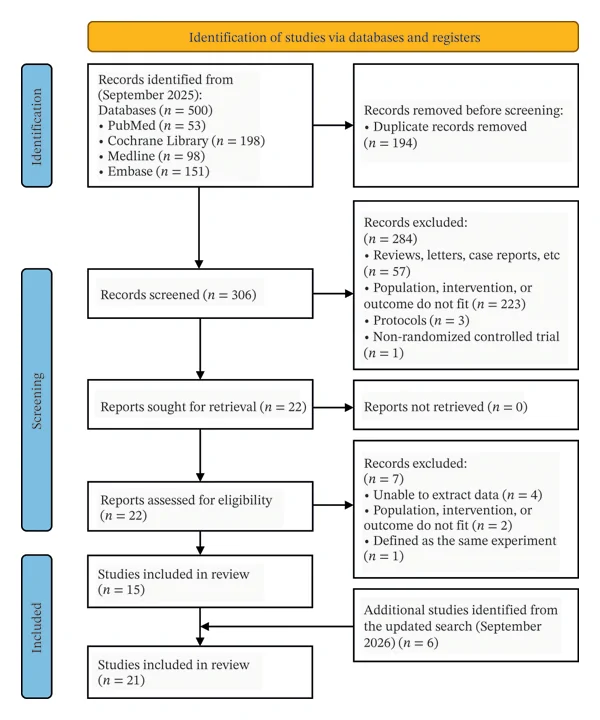
PRISMA flowchart for literature screening.

### 3.2. Study Characteristics

The basic characteristics of the included trials are summarized in Supporting Table 3.

### 3.3. Meta‐Analysis

#### 3.3.1. Pain Scores

A risk of bias assessment was performed for 17 studies [9–12, 26–38] reporting pain scores. One study [12] had a baseline imbalance and two studies [26, 36] lacked allocation concealment, all of which were rated as having some concerns in domain 1 (D1). Nine studies [12, 26–30, 34, 37, 38] did not use appropriate methods for resolving missing data and were rated as having some concerns in domain 2 (D2). One study [28] did not explain the reasons for loss to follow‐up, and another study [29] had participants lost to follow‐up because of their low pain scores. Both studies showed high overall loss‐to‐follow‐up rates (≥ 10%). Therefore, they were rated as high risk in domain 3 (D3). One study [10] reported selective reporting of postoperative pain outcomes and four studies [12, 26, 31, 35] lacked registration information, all rated as having some concerns in domain 5 (D5). Overall, four studies [9, 11, 32, 33] were rated as low risk, 11 studies [10, 12, 26, 27, 30, 31, 34–38] as having some concerns, and two studies [28, 29] as high risk (Figure 2).

**FIGURE 2:**
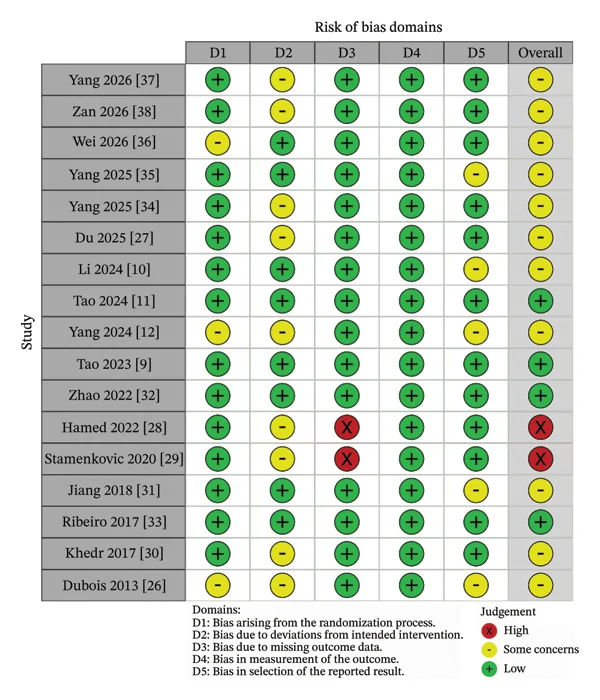
Risk of bias summary for postoperative pain [9–12, 26–38].

The meta‐analysis demonstrated that the tES group exhibited lower pain scores on postoperative day 1 compared with the control group (SMD = −0.57, 95% CI: −0.84 to −0.30, *p* < 0.01) (Figure 3). Heterogeneity was considerable (*I* ^2^ = 87%, *p* < 0.01). Sensitivity analysis revealed that the results remained stable after excluding individual studies sequentially. No obvious publication bias was detected (*p* = 0.121).

**FIGURE 3:**
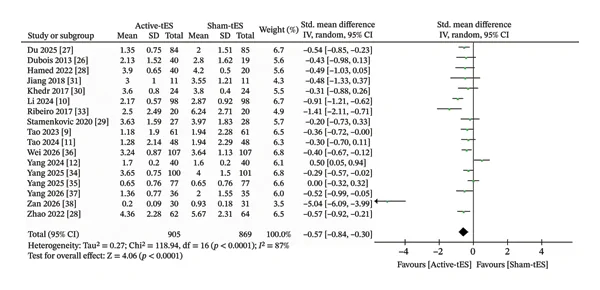
Forest plot of postoperative pain on day 1 [9–12, 26–38].

For other time points, the tES group showed lower pain scores on postoperative day 2 (SMD = −0.88, 95% CI: −1.32 to −0.44, *p* < 0.01) and postoperative day 3 (SMD = −0.75, 95% CI: −1.18 to −0.32, *p* < 0.01). No significant reductions were observed on the day of surgery (SMD = −0.30, 95% CI: −0.67 to 0.07, *p* = 0.12), postoperative day 4 (SMD = −0.23, 95% CI: −0.56 to 0.10, *p* = 0.17), postoperative day 5 (SMD = −0.37, 95% CI: −0.78 to 0.04, *p* = 0.07), or postoperative day 7 (SMD = −0.247, 95% CI: −0.494 to 0.000, *p* = 0.050). For postoperative day 6, data were available from only a single study [33], and qualitative description suggested lower pain scores in the tES group. Another study [11] reported lower pain scores in the tES group at 1 and 3 months postoperatively. The detailed results are presented in Supporting Table 4.

#### 3.3.2. Opioid Consumption

Risk of bias was assessed for the 10 studies [26–30, 36, 37, 39–41] reporting opioid consumption. Five studies [26, 36, 39–41] lacked allocation concealment (some concerns in D1). Seven studies [26–30, 37, 41] did not use appropriate methods for resolving missing data (some concerns in D2). One study [28] did not explain the reasons for loss to follow‐up, and another study [29] had participants lost to follow‐up because of their low pain scores. Both studies had high overall loss‐to‐follow‐up rates (≥ 10%) (high risk in D3). One study [28] reported selective reporting of opioid consumption, and five studies [26, 36, 39–41] lacked registration information (some concerns in D5). Overall, eight studies [26, 27, 30, 36, 37, 39–41] were rated as having some concerns and two studies [28, 29] were rated as high risk.

The meta‐analysis showed that total opioid consumption was lower in the tES group (SMD = −0.47, 95% CI: −0.74 to −0.20, *p* < 0.01). Heterogeneity was considerable (*I* ^2^ = 68%, *p* < 0.01). Sensitivity analysis revealed that the results remained stable after excluding individual studies sequentially. No obvious publication bias was detected (*p* = 0.869).

In addition, cumulative opioid consumption was lower in the tES group during the first 2 postoperative days (SMD = −0.54, 95% CI: −0.80 to −0.29, *p* < 0.01), the first 4 postoperative days (SMD = −0.47, 95% CI: −0.91 to −0.03, *p* = 0.04), and the first 5 postoperative days (SMD = −0.49, 95% CI: −0.93 to −0.05, *p* = 0.03). No significant differences were found on postoperative day 1 (SMD = −0.10, 95% CI: −0.27 to 0.07, *p* = 0.26) or during the first 3 postoperative days (SMD = −0.48, 95% CI: −1.07 to 0.10, *p* = 0.11). The detailed results are presented in Supporting Table 4.

#### 3.3.3. Quality of Recovery

Risk of bias was assessed for the four studies [11, 12, 35, 37] reporting the quality of recovery‐15 (QoR‐15) score. One study [12] had a baseline imbalance, inappropriate missing data handling, and no registration (some concerns in D1, D2, and D5). One study [37] did not use appropriate methods for resolving missing data (some concerns in D2). Overall, two studies [11, 35] were rated as low risk and two studies [12, 37] were rated as having some concerns.

The meta‐analysis showed no significant difference in QoR‐15 scores on postoperative day 1 between the tES and control groups (SMD = 0.44, 95% CI: −0.18 to 1.05, *p* = 0.16). Heterogeneity was considerable (*I* ^2^ = 89%, *p* < 0.01). Sensitivity analysis showed that the effect size changed after excluding one study [37], indicating that the results were not stable. No obvious asymmetry was observed in the funnel plot (Supporting Figure 1a).

QoR‐15 scores were higher in the tES group on postoperative day 3 (SMD = 0.29, 95% CI: 0.03 to 0.54, *p* = 0.03). A qualitative description from study [11] indicated improvements in QoR‐15 scores on the day of surgery and at 1 and 3 months (Supporting Table 4).

#### 3.3.4. Postoperative Delirium

Risk of bias was assessed for the six studies [9, 10, 34, 36, 38, 42] reporting the postoperative delirium incidence. Two studies [36, 42] lacked allocation concealment (some concerns in D1). Three studies [34, 38, 42] did not use appropriate methods for resolving missing data (some concerns in D2). One study [36] lacked registration information (some concerns in D5). Overall, two studies [9, 10] were rated as low risk and four studies [34, 36, 38, 42] as having some concerns.

The meta‐analysis showed tES was associated with a lower incidence of postoperative delirium (RR = 0.45, 95% CI: 0.25 to 0.79, *p* < 0.01). Heterogeneity was considerable (*I* ^2^ = 55%, *p* = 0.06). Sensitivity analysis showed that the effect size changed after excluding one study [10]. No obvious asymmetry was observed in the funnel plot (Supporting Figure 1b).

#### 3.3.5. Adverse Events

Risk of bias was assessed for the 15 studies [9–11, 26, 27, 29–32, 34–38, 42] reporting adverse events. Three studies [26, 36, 42] lacked allocation concealment (some concerns in D1). Eight studies [26, 27, 29, 30, 34, 37, 38, 42] inappropriately handled missing data (some concerns in D2). One study [29] reported a high overall loss‐to‐follow‐up rate, with some participants lost to follow‐up because of adverse surgical events (high risk in D3). Ten studies [9–11, 29, 30, 32, 34, 36, 38, 42] did not report complications during registration and two [26, 31] lacked registration (some concerns in D5). Overall, one study [35] was rated as low risk, 13 studies [9–11, 26, 27, 30–32, 34, 36–38, 42] were rated as having some concerns, and one study [29] was rated as high risk.

Twelve studies [9–11, 27, 29–32, 34, 36–38] reported postoperative nausea or vomiting. The incidence of postoperative nausea or vomiting was lower in the tES group (RR = 0.84, 95% CI: 0.75 to 0.95, *p* < 0.01). Heterogeneity was low (*I* ^2^ = 0%, *p* = 0.69). Sensitivity analysis showed that the effect size changed after excluding one study [32], indicating that the results were not stable. No obvious publication bias was detected (*p* = 0.336).

Eleven studies [9, 10, 27, 29–32, 35–38] reported dizziness or headache. No significant difference was found between groups (RR = 1.01, 95% CI: 0.70 to 1.47, *p* = 0.94). Heterogeneity was low (*I* ^2^ = 2%, *p* = 0.41). Sensitivity analysis confirmed the stability. No obvious publication bias was detected (*p* = 0.419).

Ten studies [9, 10, 26, 27, 30, 34–38] reported stimulation site discomfort. Incidence was higher in the active‐tES group (RR = 3.10, 95% CI: 1.96 to 4.90, *p* < 0.01). Heterogeneity was low (*I* ^2^ = 0%, *p* = 0.84). Sensitivity analysis confirmed the stability. Publication bias was present (*p* = 0.029).

### 3.4. Exploratory Subgroup Analyses, Meta‐Regression, and Outlier Analysis

Subgroup analyses revealed that postoperative day 1 pain scores were lower in the active‐tES group across all subgroups except one (stimulation intensity < 2 mA) (Figure 4). However, significant between‐subgroup differences were observed only when studies were stratified by the initial timing of stimulation (*I* ^2^ = 78%, *p* = 0.01). Preoperative initiation of stimulation yielded the largest effect size, followed by intraoperative initiation, whereas postoperative initiation produced the smallest effect. Meta‐regression analysis further identified the initial timing of stimulation as a significant moderator (*p* = 0.02), explaining 15.2% of the total heterogeneity, and the number of stimulation sessions was also significantly associated with treatment effect (*p* < 0.01), accounting for 70.98% of the total heterogeneity. No other factors demonstrated a significant moderating effect. Three studies [12, 33, 38] were identified as highly influential on the pooled estimate (Figure 5a–f). After removing these studies, heterogeneity decreased markedly (*I* ^2^ = 38%; *p* = 0.07), and the pooled analgesic effect remained present (SMD = −0.42; 95% CI: −0.55 to −0.29; *p* < 0.01).

**FIGURE 4:**
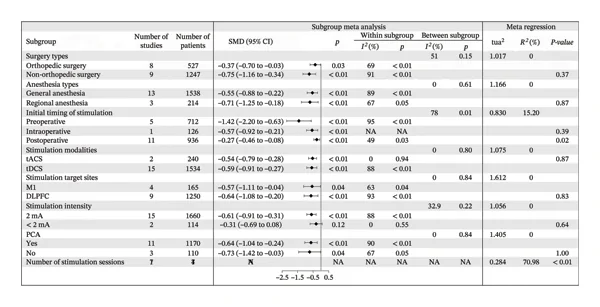
Subgroup analyses and univariate meta‐regression for postoperative pain on day 1 [9–12, 26–38].

**FIGURE 5:**
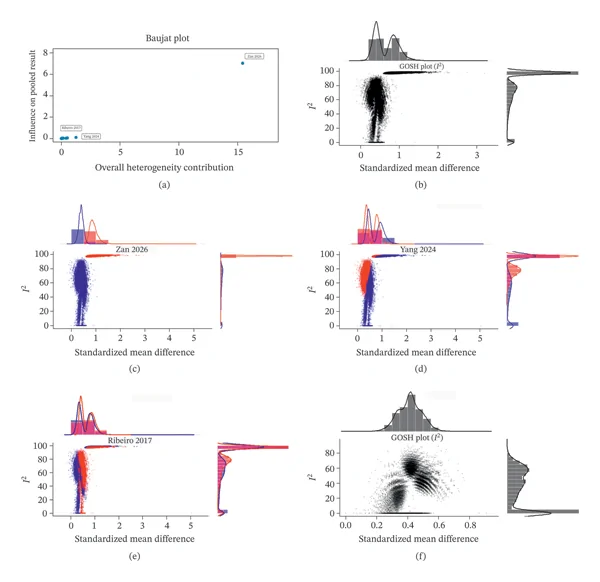
(a) Baujat plot for postoperative pain on day 1. (b) GOSH plot for postoperative pain on day 1. (c) GOSH plot after excluding the Zan 2026 study [38]. (d) GOSH plot after excluding the Yang 2024 study [12]. (e) GOSH plot after excluding the Ribeiro 2017 study [33]. (f) GOSH plot after excluding all three studies [9–12, 26–38].

Subgroup analyses showed that the active‐tES group had lower total opioid consumption in most subgroups (Supporting Figure 2). The effect size was not statistically significant in several subgroups with a small number of included studies, including trials using regional anesthesia, tACS, stimulation intensity < 0.2 mA, and those without PCA. The subgroup difference stratified by the initial timing of stimulation was marginally significant (*I* ^2^ = 71.1%, *p* = 0.06). Stimulation initiated preoperatively yielded a larger effect size than stimulation initiated postoperatively. Meta‐regression indicated that none of the predefined moderators exhibited a significant moderating effect. Stimulation modality explained 22.59% of total heterogeneity and initial timing of stimulation explained 2.71% of total heterogeneity, yet the moderating effects of these two variables failed to reach statistical significance. Furthermore, when performing subgroup analysis for stimulation targets, only five studies were eligible for inclusion. Within‐study heterogeneity was fully eliminated (*R* ^2^ = 100%); however, the moderating effect was nonsignificant. After excluding the two most influential studies [27, 28], heterogeneity decreased substantially (*I* ^2^ = 31%, *p* = 0.18) and the pooled effect remained statistically significant (SMD = −0.50; 95% CI: −0.72 to −0.27; *p* < 0.01) (Supporting Figure 3a–e).

Owing to the limited number of included studies, exploratory subgroup analyses, meta‐regression, and outlier analysis were not performed for the quality of recovery and postoperative delirium outcomes.

### 3.5. TSA and Certainty of Evidence

For postoperative day 1 pain scores (Figure 6) and postoperative delirium (Supporting Figure 4c), the cumulative Z‐curve crossed both the conventional statistical boundary and the TSA sequential monitoring boundary and achieved the pre‐specified required information size (RIS). This finding indicates that the TSA supports that perioperative tES reduces postoperative day 1 pain scores and the incidence of postoperative delirium. The certainty of evidence for these two outcomes was rated moderate and low, respectively (Supporting Table 5). For total opioid consumption, the cumulative Z‐curve crossed the TSA sequential monitoring boundary but failed to reach the prespecified RIS (Supporting Figure 4a). These findings suggested that the cumulative evidence achieved statistical significance, while the sample size was insufficient to meet the ideal information threshold. And the certainty of evidence for this outcome was rated as low (Supporting Table 5). For postoperative quality of recovery on day 1, the cumulative Z‐curves neither crossed the conventional statistical boundaries nor reached the pre‐specified RIS, indicating that current cumulative evidence is insufficient to determine whether perioperative tES is associated with early postoperative quality of recovery (Supporting Figure 4b). The certainty of evidence for the outcome was graded as very low (Supporting Table 5). Regarding safety outcomes, the GRADE certainty of evidence was low for postoperative nausea or vomiting, and moderate for postoperative dizziness or headache and stimulation site discomfort (Supporting Table 5).

**FIGURE 6:**
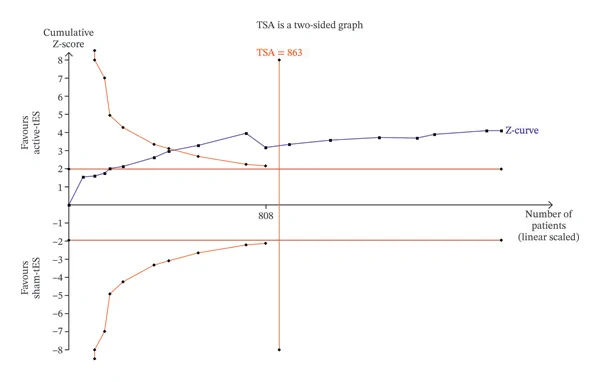
TSA plot for postoperative pain on day 1 [9–12, 26–38].

## 4. Discussion

This systematic review and meta‐analysis included 21 RCTs involving 2186 patients and evaluated the effects of perioperative tES on several clinically relevant postoperative outcomes. The main findings showed that perioperative tES was associated with lower postoperative day 1 pain scores and lower total postoperative opioid consumption, and may also be associated with a lower incidence of postoperative delirium. In contrast, tES did not show a clear benefit in improving quality of recovery on postoperative day 1. Active tES was not associated with an increased incidence of postoperative dizziness or headache, but was associated with a higher incidence of stimulation site discomfort. Importantly, substantial heterogeneity was observed for the main efficacy outcomes, and the certainty of evidence varied from very low to moderate across outcomes.

Influenced by the concept of multimodal postoperative analgesia, neuromodulation has gradually attracted clinical attention in the perioperative period. For example, transcutaneous electrical nerve stimulation, as a peripheral nerve stimulation technique, may contribute to pain control by modulating sensory nerve activity at the peripheral and spinal levels [43]. In contrast, tES delivers current via scalp electrodes to regulate cortical excitability and related brain network activity within the central nervous system [44]. A 2024 systematic review and meta‐analysis including 40 studies found that transcutaneous electrical nerve stimulation reduced postoperative pain at rest, pain during coughing, and morphine consumption, and was also associated with a lower incidence of some postoperative adverse events [45]. These findings provide broader support for the potential role of neuromodulation as an adjunct to multimodal postoperative pain management.

A previous meta‐analysis mainly evaluated changes in postoperative pain and opioid consumption in patients receiving tDCS under general anesthesia [13]. Compared with previous studies, we included both tDCS and tACS and considered different perioperative clinical settings, stimulation timings, and postoperative outcomes. This further extends the scope of evidence for tES use in the perioperative setting.

The pooled results showed that tES was associated with lower pain scores on postoperative day 1. However, the included studies used different pain assessment scales, assessment conditions, and assessment time points. Although VAS and NRS can be reasonably standardized, details of pain assessment were not reported in full in some studies. Therefore, the SMD in this meta‐analysis should be interpreted as an average treatment effect across similar pain assessment methods rather than as a direct effect of tES on a single fully standardized pain outcome. Furthermore, although our meta‐analysis showed lower postoperative day 1 pain scores in the tES group, the between‐group difference (mean difference = −0.53) did not reach the minimal clinically important difference [46]. This suggests that tES should be considered an adjunctive component of postoperative analgesia rather than a replacement for pharmacological analgesics in patients undergoing elective surgery.

The analgesic effects of tES may involve several mechanisms. Previous studies have shown that stimulation of the M1 can enhance the endogenous analgesic system function by activating descending inhibitory pathways, including the thalamocortical‐brainstem network. In contrast, stimulation of the DLPFC may reduce the subjective perception of pain by modulating emotion, attention, and cognitive processing of pain [47, 48]. These findings provide a theoretical basis for the use of tES in perioperative pain management. Experimental studies have also suggested that preoperative tDCS may influence the development of persistent pain and hyperalgesia, providing a theoretical basis for the possibility that stimulation before surgery may affect central nociceptive processing [49, 50]. In addition, one study demonstrated that tACS targeting the left DLPFC could increase the heat pain threshold in healthy individuals, thereby producing an analgesic effect [51].

The substantial heterogeneity observed in postoperative pain and opioid consumption is an important issue that warrants careful consideration. The included studies differed substantially in surgical type, anesthetic type, stimulation modality, stimulation target, stimulation timing, stimulation intensity, number of sessions, and background analgesic strategies. These differences may all affect the baseline level of postoperative pain and patients’ responses to neuromodulatory treatment. Exploratory subgroup analyses and meta‐regression suggested that the initial timing of stimulation and number of sessions may account for part of the observed heterogeneity. However, these factors explained only part of the heterogeneity, and several other clinical variables did not show statistically significant associations. Importantly, although PCA use was included as a potential moderator, broader aspects of analgesic management, including specific drugs, doses, routes of administration, and multimodal analgesic combinations, could not be reliably analyzed quantitatively because of inconsistent reporting. Furthermore, we found that a small number of studies may have made a disproportionate contribution to the observed statistical heterogeneity. However, excluding these studies had no substantial impact on our findings, indicating that our results were robust to the influence of individual studies.

Subgroup analyses showed lower postoperative day 1 pain scores in the tES group across the examined strata defined by surgical type, anesthesia modality, stimulation modalities, initial timing of stimulation, stimulation target sites, and use of PCA. However, this difference was not observed in the subgroup with low stimulation intensity (< 2 mA), which included only two studies [26, 29]. Similarly, in some small subgroups, the between‐group difference in total opioid consumption no longer reached statistical significance. Research on perioperative tES analgesia remains scarce in several scenarios: regional anesthesia, preoperative or intraoperative stimulation (early intervention), stimulation intensity < 2 mA, and tACS. Accordingly, these subgroups included only a small number of studies, and these findings may not represent the true underlying effect. One notable finding from the exploratory analyses was that the initial timing of stimulation and the number of stimulation sessions may be associated with the magnitude of the postoperative analgesic effect. Preventive intervention may reduce postoperative pain more effectively than treatment initiated after tissue injury by preemptively modulating the excitatory–inhibitory balance within the central nociceptive networks, influencing neuroplasticity‐related factors, and inducing neuroimmune regulatory effects [33, 52]. This finding is consistent with the concept of preventive analgesia. The association between the number of stimulation sessions and treatment effect may also suggest a cumulative neuromodulatory effect of repeated stimulation. A previous meta‐analysis in chronic pain similarly reported an association between the number of stimulation sessions and treatment response [53]. However, these findings do not establish that preoperative stimulation or a greater number of stimulation sessions is superior to other approaches. Both subgroup analyses and meta‐regression were based on study‐level characteristics and are susceptible to confounding.

No statistically significant difference in QoR‐15 scores was observed on postoperative day 1, and the certainty of evidence for this outcome was very low. This finding is important because improvement in pain does not necessarily translate directly into better overall postoperative recovery. Quality of recovery is a multidimensional outcome that includes physical comfort, physical independence, psychological status, cognition, and other aspects of recovery [54]. Although some studies reported improvements in QoR‐15 scores at later time points with tES, the number of relevant studies was small and substantial heterogeneity was present. In addition, sensitivity analysis suggested that the postoperative day 1 result was influenced by individual studies. Therefore, current evidence is insufficient to determine whether perioperative tES improves overall early postoperative quality of recovery. Future studies should include validated quality of recovery measures as prespecified important outcomes rather than assuming that improvement in pain necessarily translates into better overall recovery.

The association between tES and a lower incidence of postoperative delirium is of potential clinical importance. Most existing studies have targeted the DLPFC to investigate the effects of tES on postoperative delirium. The underlying rationale is that the DLPFC serves as a key brain region involved in cognitive regulation [55]. Previous studies in healthy participants and patients with neuropsychiatric disorders have demonstrated that tES targeting the DLPFC can improve cognitive performance, including memory, attention, and perceptual processing [56, 57]. However, the available clinical evidence remains limited. Only six RCTs in our review evaluated postoperative delirium, and sensitivity analysis showed that the pooled result became nonsignificant after excluding one study. Therefore, the certainty of evidence for this outcome was rated as low.

The available evidence did not suggest that tES increased the risk of postoperative dizziness or headache, and the certainty of evidence for this outcome was moderate. The most common potential adverse effect was local discomfort at the stimulation site, which was generally reported as mild and transient. A previous meta‐analysis of transcutaneous electrical acupoint stimulation also suggested that electrical stimulation may cause mild discomfort that usually resolves spontaneously [58]. The lower incidence of postoperative nausea or vomiting is also noteworthy. However, this association showed some instability in sensitivity analysis, and one study [32] recruiting patients undergoing cesarean delivery contributed substantially to the overall effect. This population has inherently high baseline risk of postoperative nausea or vomiting, which may account for the prominent beneficial effect observed. Therefore, until more evidence becomes available, this finding should not be interpreted as evidence of a clear direct antiemetic effect of tES.

The methodological quality of the included studies is an important limitation of this review. Most studies had at least some concerns regarding allocation concealment, missing data, or prospective trial registration, and some studies were judged to have a high risk of bias for specific outcomes. Together with unexplained between‐study heterogeneity, the evidence merits downgrading even if the meta‐analysis identifies a statistically significant difference and TSA indicates a positive effect.

Although previous meta‐analyses have confirmed that specific tES modalities yield analgesic effects and reduce opioid consumption in specific surgical procedures and anesthesia settings, our study provides new insights into the existing tES literature by comprehensively evaluating its efficacy for postoperative pain management across diverse clinical scenarios. This approach enables a more generalizable assessment of tES for postoperative pain control and offers valuable references for investigations applicable to a wide range of clinical settings. The present study comprehensively examined a set of postoperative outcomes, including pain score, opioid consumption, quality of recovery, postoperative delirium, and specific adverse events. This comprehensive analytical approach allows for a deeper understanding of the multifaceted impacts of tES on postoperative recovery.

The strength of evidence in this study is limited by the following factors. (1) The overall risk of bias among the included studies was relatively high, particularly in the domains of allocation concealment, handling of missing outcome data, and transparency of trial registration. (2) Substantial heterogeneity was observed across studies. Although subgroup analyses and meta‐regression partially explained some of this variation, uncontrolled confounding factors likely remained. In addition, several subgroups contained only a small number of studies, so the effect size estimates may be imprecise. Furthermore, subgroup analyses and meta‐regression were exploratory in nature and their results should therefore be interpreted with caution. (3) The number of included studies was small for several outcomes, and publication bias was assessed only using funnel plots, which may limit the ability to detect publication bias. (4) TSA considers only the accumulation of information, without accounting for the quality of the original trials; its results may therefore be relatively optimistic. (5) Some outcomes were rated as having low or very low certainty according to the GRADE framework. (6) Only a few studies reported follow‐up data on medium‐ and long‐term postoperative outcomes.

Future research should: (1) conduct large‐sample, multicenter, standardized RCTs to reduce the uncertainty in judging evidence certainty that arises from the methodological quality of original trials; (2) expand research covering different surgical types, regional anesthesia, tACS, and early intervention to clarify the generalizability of the analgesic efficacy of tES across diverse clinical scenarios; (3) compare the advantages and disadvantages of different stimulation protocols in the perioperative setting (including stimulation modality, target site, stimulation parameters, timing of intervention initiation, and number of sessions); (4) expand the range of assessed outcomes for elective surgical patients receiving tES to clarify the multifaceted postoperative effects of tES in surgical patients; (5) apply neuroimaging techniques to explore the underlying mechanisms of action; and (6) expand the follow‐up duration to assess chronic postsurgical pain and long‐term quality of recovery.

In summary, tES, as a noninvasive and repeatable neuromodulation technique, may serve as an adjuvant approach for perioperative analgesia and opioid sparing, while showing potential for preventing postoperative delirium. However, the effect of tES on postoperative quality of recovery remains unclear. Mild transient local discomfort is a common adverse reaction, but its overall safety profile is favorable. Given the limited quantity and quality of existing studies, further large‐scale, high‐quality RCTs are warranted to validate these findings and to optimize stimulation protocols.

**Nomenclature**
CIs: confidence intervals
D: domain
DLPFC: dorsolateral prefrontal cortex
GDT: Guideline Development Tool
GOSH: Graphical display of study heterogeneity
GRADE: Grading of Recommendations Assessment, Development, and Evaluation
IQR: interquartile ranges
M1: primary motor cortex
MMEs: morphine milligram equivalents
NRS: Numeric Rating Scale
PCA: patient‐controlled analgesia
PRISMA: Preferred Reporting Items for Systematic Reviews and Meta‐Analyses
QoR‐15: quality of recovery‐15
RCTs: randomized controlled trials
RIS: required information size
RoB 2: Risk of Bias 2
RR: risk ratio
SD: standard deviations
SMD: standardized mean difference
tACS: transcranial alternating current stimulation
tDCS: transcranial direct current stimulation
tES: transcranial electrical stimulation
TSA: trial sequential analysis
VAS: Visual Analogue Scale

## Author Contributions

Zehao Liu, Yixin Zhang, Yangyang Li, Wenjie Mao, and Qing Zhong: conceptualization, methodology, and design; Yixin Zhang, Yangyang Li, Dezhou Jiang, Funing Liu, Chunmei Wu, Xiaolan Li, Suying Li, Wenjie Mao, and Qing Zhong: acquisition and analysis of data; Zehao Liu: software, visualization, and writing–original draft; Qing Zhong: funding acquisition. Zehao Liu, Yixin Zhang, and Yangyang Li are the co‐first authors.

## Funding

This research was funded by the Jianyang People’s Hospital Hospital‐Level Research Project (JYk202483, JY202569), the Sichuan International Medical Exchange & Promotion Association (L20230410015, L20231210001), the Medical Research Project of Chengdu Health Commission (2024164), the Sichuan Medical Association Medical Research Project (S20250003), and the Health Commission of Chengdu‐Chengdu Medical College 2025 Commission‐University Joint Innovation Fund Project (WXLHCXJJ25‐56).

## Disclosure

The funders had no influence on the outcome of the study. All the authors read, revised, and approved the final version.

## Ethics Statement

The authors have nothing to report.

## Conflicts of Interest

The authors declare no conflicts of interest.

## Supporting Information

Additional supporting information can be found online in the Supporting Information section.

## Supporting information


**Supporting Information 1** Supporting Table 1 shows the full search strategies used for each database (PubMed, Cochrane Library, MEDLINE, and Embase). Supporting Table 2 shows the characteristics of pain assessment for pain outcomes in the included studies. Supporting Table 3 shows the characteristics of the 21 included studies, including country, surgery type, anesthesia method, sample size, age, intervention details, and outcomes. Supporting Table 4 shows the meta‐analysis results for pain scores, cumulative opioid consumption, and quality of recovery at different postoperative time points (days and months). Supporting Table 5 shows the GRADE certainty of evidence assessment for all outcomes, including pain scores, opioid consumption, quality of recovery, delirium, and adverse events.

**Supporting Information 2** Supporting Figure 1 shows the funnel plots for postoperative quality of recovery on day 1 and postoperative delirium. Supporting Figure 2 shows the subgroup analyses and univariate meta‐regression for total opioid consumption. Supporting Figure 3 shows the Baujat plot and GOSH plots for postoperative total opioid consumption. Supporting Figure 4 shows the TSA plots for postoperative total opioid consumption, postoperative quality of recovery on day 1, and postoperative delirium.

## Data Availability

The data that support the findings of this study are available from the corresponding author upon reasonable request.
