# Supplementary material for: Efficacy and Safety of Perioperative Transcranial Electrical Stimulation for Postoperative Pain and Related Outcomes: A Systematic Review and Meta‐Analysis With Trial Sequential and Subgroup Analyses
Source: Pain Res Manag. 2026 Oct 23;2026:4293899. doi: 10.1155/prm/4293899 (PMC13693405; doi:10.1155/prm/4293899)
Supplement: Supplementary file 1 [file PRM-2026-4293899-s001.docx]

**Supplemental Table 1.** The full search strategy for each database.

|  | **Search strategy** |
| --- | --- |
| **PubMed** | ((((((((((transcranial electrical stimulation[MeSH Terms]) OR (transcranial electrical stimulation[Title/Abstract])) OR (transcranial direct current stimulation[Title/Abstract])) OR (transcranial alternating current stimulation[Title/Abstract])) OR (transcranial random noise stimulation[Title/Abstract])) OR (tES[Title/Abstract])) OR (tDCS[Title/Abstract])) OR (tACS[Title/Abstract])) OR (tRNS[Title/Abstract])) AND ((((((((((((((postoperative period[MeSH Terms]) OR (postoperative period[Title/Abstract])) OR (postoperative periods[Title/Abstract])) OR (postoperative[Title/Abstract])) OR (post-operative[Title/Abstract])) OR (after surgery[Title/Abstract])) OR (following surgery[Title/Abstract])) OR (post surgical[Title/Abstract])) OR (post-surgical[Title/Abstract])) OR (perioperative period[MeSH Terms])) OR (perioperative period[Title/Abstract])) OR (perioperative periods[Title/Abstract])) OR (perioperative[Title/Abstract])) OR (peri-operative[Title/Abstract]))) AND (((((((((((random allocation[MeSH Terms]) OR (random allocation[Title/Abstract])) OR (allocation, random[Title/Abstract])) OR (randomized controlled trial[Title/Abstract])) OR (randomized controlled trials[Title/Abstract])) OR (random[Title/Abstract])) OR (randomized[Title/Abstract])) OR (randomised[Title/Abstract])) OR (randomly[Title/Abstract])) OR (randomization[Title/Abstract])) OR (RCT[Title/Abstract])) |
| **Cochrane Library** | #1: MeSH descriptor: [Transcranial Direct Current Stimulation] explode all trees  #2: (transcranial electrical stimulation):ti,ab,kw OR (transcranial direct current stimulation):ti,ab,kw OR (transcranial alternating current stimulation):ti,ab,kw OR (transcranial random noise stimulation):ti,ab,kw OR (tES):ti,ab,kw OR (tDCS ):ti,ab,kw OR (tACS):ti,ab,kw OR (tRNS):ti,ab,kw  #3: #1 OR #2  #4: MeSH descriptor: [Postoperative Period] explode all trees  #5: MeSH descriptor: [Perioperative Period] explode all trees  #6: (postoperative period):ti,ab,kw OR (postoperative periods):ti,ab,kw OR (postoperative):ti,ab,kw OR (post-operative):ti,ab,kw OR (after surgery):ti,ab,kw OR (following surgery):ti,ab,kw OR (post surgical):ti,ab,kw OR (post-surgical):ti,ab,kw OR (perioperative period):ti,ab,kw OR (perioperative periods):ti,ab,kw OR (perioperative):ti,ab,kw OR (peri-operative):ti,ab,kw  #7: #4 OR #5 OR #6  #8: MeSH descriptor: [Random Allocation] explode all trees  #9: (random allocation):ti,ab,kw OR (allocation, random):ti,ab,kw OR (randomized controlled trial):ti,ab,kw OR (randomized controlled trials):ti,ab,kw OR (random):ti,ab,kw OR (randomized):ti,ab,kw OR (randomised):ti,ab,kw OR (randomly):ti,ab,kw OR (randomization):ti,ab,kw OR (RCT ):ti,ab,kw  #10: #8 OR #9  #11: #3 AND #7 AND #10 |
| **Medline** | #1: TS=(transcranial electrical stimulation) OR TS=(transcranial direct current stimulation) OR TS=(transcranial alternating current stimulation) OR TS=(transcranial random noise stimulation) OR TS=(tES) OR TS=(tDCS ) OR TS=(tACS) OR TS=(tRNS)  #2: TS=(postoperative period) OR TS=(postoperative periods) OR TS=(postoperative) OR TS=(post-operative) OR TS=(after surgery) OR TS=(following surgery) OR TS=(post surgical) OR TS=(post-surgical) OR TS=(perioperative period) OR TS=(perioperative periods) OR TS=(perioperative) OR TS=(peri-operative)  #3: TS=(random allocation) OR TS=(allocation, random) OR TS=(randomized controlled trial) OR TS=(randomized controlled trials) OR TS=(random) OR TS=(randomized) OR TS=(randomised) OR TS=(randomly) OR TS=(randomization) OR TS=(RCT)  #4: #1 AND #2 AND #3 |
| **Embase** | #11: #3 AND #7 AND #10  #10: #8 OR #9  #9: 'transcranial electrical stimulation':ti,ab,kw OR 'transcranial direct current stimulation':ti,ab,kw OR 'transcranial alternating current stimulation':ti,ab,kw OR 'transcranial random noise stimulation':ti,ab,kw OR 'tes':ti,ab,kw OR 'tdcs':ti,ab,kw OR 'tacs':ti,ab,kw OR 'trns':ti,ab,kw  #8: 'transcranial electrical stimulation'/exp  #7: #4 OR #5 OR #6  #6: 'postoperative period':ti,ab,kw OR 'postoperative periods':ti,ab,kw OR 'postoperative':ti,ab,kw OR 'post-operative':ti,ab,kw OR 'after surgery':ti,ab,kw OR 'following surgery':ti,ab,kw OR 'post surgical':ti,ab,kw OR 'post-surgical':ti,ab,kw OR 'perioperative period':ti,ab,kw OR 'perioperative periods':ti,ab,kw OR 'perioperative':ti,ab,kw OR 'peri-operative':ti,ab,kw  #5: 'perioperative period'/exp  #4: 'postoperative period'/exp  #3: #1 OR #2  #2: 'random allocation':ti,ab,kw OR 'allocation, random':ti,ab,kw OR 'randomized controlled trial':ti,ab,kw OR 'randomized controlled trials':ti,ab,kw OR 'random':ti,ab,kw OR 'randomized':ti,ab,kw OR 'randomised':ti,ab,kw OR 'randomly':ti,ab,kw OR 'randomization':ti,ab,kw OR 'rct':ti,ab,kw  #1: 'random allocation'/exp |

**Supplemental Table 2.** Characteristics of pain assessment for pain outcomes in the included studies.

| **Study** | **Pain scale** | **Pain assessment condition** | **Pain assessment time‑points** |
| --- | --- | --- | --- |
| Yang 2026 [37] | NRS | At rest; on movement | 2 hours, 6 hours, 12 hours, 24 hours and 48 hours after surgery |
| Zan 2026 [38] | NRS | At rest; active arm abduction (up to 90 degrees) on the surgical side | postoperative days 1 to 5 |
| Wei 2026 [36] | NRS | Unclear | postoperative days 1 to 3; 1 week after surgery; 1 month after surgery |
| Yang 2025 [35] | NRS | Unclear | in the post‑anesthesia care unit; 24 hours after surgery |
| Yang 2025 [34] | NRS | Unclear | afternoon of postoperative day 1 |
| Du 2025 [27] | NRS | At rest; on movement | 24 hours and 72 hours after surgery |
| Li 2024 [10] | NRS | Unclear | 2 hours after surgery; postoperative days 1 to 3 |
| Tao 2024 [11] | NRS | At rest; on movement | 2 hours after surgery; postoperative day 1; 1 month and 3 months after surgery |
| Yang 2024 [12] | NRS | Unclear | night of postoperative day 0; night of postoperative day 1 |
| Tao 2023 [9] | NRS | At rest; on movement | 2 hours after surgery; morning and afternoon on postoperative days 1 to 3 |
| Zhao 2022 [32] | VAS | Unclear | 24 hours after surgery |
| Hamed 2022 [28] | VAS | At rest; during walking for 10 meters | postoperative days 1 to 3 |
| Stamenkovic 2020 [29] | VAS | At rest; on movement; during coughing | morning on postoperative days 1 to 5 |
| Jiang 2018 [31] | NRS | Unclear | morning of postoperative day 1 |
| Ribeiro 2017 [33] | VAS | At rest; during walking | postoperative days 0 to 7 |
| Khedr 2017 [30] | VAS | Unclear | 2 hours after surgery; morning on postoperative days 1 to 3 |
| Dubois 2013 [26] | VAS | At rest; during moving in the bed | postoperative days 1 to 2 |

**NRS**: Numeric Rating Scale; **VAS**: Visual Analogue Scale

**Supplemental Table 3.** Characteristics of the included studies.

| **Study** | **Country** | **Surgery** | **Primary anesthesia method** | **Sample size (A/S/Total)** | | | **Age**  **(A/S)**  **(Mean/median)** | | **Intervention** | | | |
| --- | --- | --- | --- | --- | --- | --- | --- | --- | --- | --- | --- | --- |
|  |  |  |  |  |  |  |  |  | **Site (A/S)** | | **Parameters (A/S)** | |
| Antonenko 2026 [42] | Germany | Elective surgery including vascular, abdominal, thoracic, urological, gynecological, and spinal procedures | General anesthesia | 68 | 74 | 225 | 73* | 73* | Posterior parietal cortex | Posterior parietal cortex  OR  Anode: the left DLPFC  Cathode: the right supraorbital frontal lobe | Begin: a 10-s ramp-up phase  Maintain: 9.5 Hz, 2 mA, 20 min  End: a 10­s ramp-down phase | Begin: a 10-s ramp-­up phase + 9.5 Hz, 2 mA, 30 s OR 2 mA, 30 s + a 10-s ramp-down phase  Maintain: none  End: none |
|  |  |  |  | 83 |  |  | 73* |  | Anode: the left DLPFC  Cathode: the right supraorbital frontal lobe |  | Begin: a 10-s ramp-up phase  Maintain:2 mA, 20 min  End: a 10­s ramp-down phase |  |
| Yang 2026 [37] | China | Video-assisted thoracic surgery | General anesthesia | 36 | 36 | 72 | 55.4 | 59.2* | S1 | S1 | Begin: a 30-s ramp­up phase  Maintain: 10 Hz, 2 mA, 19 min  End: a 30­s ramp-down phase | Begin: a 30-s ramp­up phase  Maintain: none, 19 min  End: a 30­s ramp-down phase |
| Zan 2026 [38] | China | Breast cancer surgery | General anesthesia | 32 | 32 | 64 | 54.00* | 51.93* | Anode: the left DLPFC  Cathode: the right DLPFC | Anode: the left DLPFC  Cathode: the right DLPFC | Begin: a 15-s ramp-up phase  Maintain: 2 mA, 20 min  End: a 15­s ramp-down phase | Begin: a 15-s ramp­up phase + a 15-s ramp-down phase  Maintain: none  End: none |
| Wei 2026 [36] | China | Abdominal cancer surgery | General anesthesia | 107 | 107 | 214 | 70 | 70 | Anode: the left DLPFC  Cathode: the right supraorbital frontal lobe | Anode: the left DLPFC  Cathode: the right supraorbital frontal lobe | 2mA, 20min | Unclear |
| Yang 2025 [35] | China | Transurethral resection of the prostate | General anesthesia | 77 | 77 | 154 | 69 | 70 | Anode: the left DLPFC  Cathode: the right supraorbital frontal lobe | Anode: the left DLPFC  Cathode: the right supraorbital frontal lobe | Begin: a 30-s ramp-up phase  Maintain: 2 mA, 10 min  End: a 30­s ramp-down phase | Begin: a 30-s ramp­up phase + a 30-s ramp-down phase  Maintain: none  End: none |
| Yang 2025 [34] | China | Laparoscopic surgery | General anesthesia | 110 | 110 | 220 | 72* | 72* | Anode: the left DLPFC  Cathode: the right supraorbital frontal lobe | Anode: the left DLPFC  Cathode: the right supraorbital frontal lobe | Begin: a 30-s ramp-up phase  Maintain: 2 mA, 19 min  End: a 30­s ramp-down phase | Begin: a 30-s ramp­up phase  Maintain: none, 19 min  End: a 30­s ramp-down phase |
| Du 2025 [27] | China | Gynecological laparoscopic surgery | General anesthesia | 88 | 88 | 176 | 45.0 | 47.5 | The PFC | The PFC | Begin: a 30-s ramp­up phase  Maintain: 7 Hz, 2 mA, 20 min  End: a 30­s ramp-down phase | Begin: a 30-s ramp­up phase  Maintain: none, 20 min  End: a 30­s ramp-down phase |
| Li 2024 [10] | China | Laparoscopic radical resection of colorectal cancer | General anesthesia | 98 | 98 | 196 | 63.5 | 63.5 | Anode: the left DLPFC  Cathode: the right DLPFC | Anode: the left DLPFC  Cathode: the right DLPFC | Begin: a 30-s ramp­up phase  Maintain: 2 mA, 20 min  End: a 30­s ramp-down phase | Begin: a 30-s ramp­up phase  Maintain: none, 20 min  End: a 30­s ramp-down phase |
| Tao 2024 [11] | China | THA or TKA | General anesthesia | 48 | 48 | 96 | 70 | 71 | Anode: the left DLPFC  Cathode: the right orbitofrontal area | Anode: the left DLPFC  Cathode: the right orbitofrontal area | Begin: a 30-s ramp­up phase  Maintain: 2 mA, 20 min  End: a 30­s ramp-down phase | Begin: a 30-s ramp­up phase  Maintain: none, 20 min  End: a 30­s ramp-down phase |
| Yang 2024 [12] | China | THA or TKA | General anesthesia | 47 | 45 | 92 | 70.3* | 73.1* | Anode: the left DLPFC  Cathode: the right supraorbital frontal lobe | Anode: the left DLPFC  Cathode: the right supraorbital frontal lobe | Begin: a 30-s ramp­up phase  Maintain: 2 mA, 20 min  End: a 30­s ramp-down phase | Begin: a 30-s ramp­up phase + a 30­s ramp-down phase  Maintain: none, 20 min  End: none |
| Tao 2023 [9] | China | THA or TKA | General anesthesia | 61 | 61 | 122 | 70 | 72 | Anode: the left DLPFC  Cathode: the right orbitofrontal area | Anode: the left DLPFC  Cathode: the right orbitofrontal area | Begin: a 30-s ramp­up phase  Maintain: 2 mA, 20 min  End: a 30­s ramp-down phase | Begin: a 30-s ramp­up phase  Maintain: none, 20 min  End: a 30­s ramp-down phase |
| Zhao 2022 [32] | China | Cesarean section | Regional anesthesia | 62 | 64 | 126 | 30.82 | 30.39 | DLPFC | DLPFC | Maintain: 120 Hz, 250 µs, 2 mA, 20 min | Maintain: 120 Hz, 250 µs, 2 mA, 30 s |
| Hamed 2022 [28] | Egypt | Lumbar discectomy and/or laminectomy | General anesthesia | 22 | 23 | 67 | 45* | 41* | Anode: the M1  Cathode: the contralateral arm (extracephalic) | Anode: the M1  Cathode: the contralateral arm (extracephalic) | Maintain: 2 mA, 20 min | Begin: 2 mA, 30 s  Maintain: none, 19 min  End: 2 mA, 30 s |
|  |  |  |  | 22 |  |  | 42* |  | Anode: the left DLPFC  Cathode: the contralateral arm (extracephalic) |  |  |  |
| Stamenkovic 2020 [29] | Serbia | Thoracotomy for lung cancer | General anesthesia | 31 | 31 | 62 | 61.44* | 61.89* | Anode: the left M1  Cathode: the right supraorbital area | Anode: the left M1  Cathode: the right supraorbital area | Begin: a 10-s ramp­up phase  Maintain: 1.2 mA, 20 min  End: a ramp-down phase | Begin: a 10-s ramp­up phase + 1.2 mA, 60 s + a ramp-down phase  Maintain: none, 20 min  End: ramped up slowly and then turned off more quickly |
| Jiang 2018 [31] | China | Lumbar spine surgery | Unclear | 11 | 11 | 22 | 47.9 | 50.5 | Anode: the M1  Cathode: the contralateral supraorbital area | Anode: the M1  Cathode: the contralateral supraorbital area | Begin: a 10-s ramp­up phase  Maintain: 2 mA, 20 min  End: a 10­s ramp-down phase | Begin: a 10-s ramp­up phase + a 10­s ramp-down phase  Maintain: none, 20 min  End: a 10-s ramp­up phase + a 10­s ramp-down phase |
| Ribeiro 2017 [33] | Brazil | Hallux valgus surgery | Regional anesthesia | 20 | 20 | 40 | 48.36 | 46.00 | Anode: the left M1  Cathode: the right supraorbital area | Anode: the left M1  Cathode: the right supraorbital area | Maintain: 2 mA, 20 min | Maintain: 2 mA, 30 s |
| Khedr 2017 [30] | Egypt | TKA | Regional anesthesia | 25 | 25 | 50 | 55.2* | 54.6* | Anode: the M1  Cathode: the ipsilateral arm (extra-cephalic) | Anode: the M1  Cathode: the ipsilateral arm (extra-cephalic) | Begin: a 15-s ramp­up phase  Maintain: 2 mA, 20 min  End: a 15­s ramp-down phase | Begin: a 15-s ramp­up phase  Maintain: none, 20 min  End: a 15­s ramp-down phase |
| Borckardt 2017 [39] | United States of America | TKA | General anesthesia | 16 | 15 | 58 | 57.79 | 62.53 | Anode: the left DLPFC  Cathode: the sensory cortex | Anode: the left temporal-occipital junction  Cathode: the medial anterior pre-motor area | Begin: a ramp­up phase  Maintain: 2 mA, 20 min  End: a ramp­ down phase | Begin: a ramp­up phase  Maintain: 2 mA, 20 min  End: a ramp­ down phase |
|  |  |  |  | 14 | 13 |  | 60.19 | 64.54 | Anode: the motor cortex  Cathode: the right DLPFC | Anode: left DLPFC  Cathode: the sensory cortex  OR  Anode: the motor cortex  Cathode: the right DLPFC |  | Begin: a ramp­up phase  Maintain: 2 mA, 30 s  End: a ramp­ down phase |
| Glaser 2016 [40] | United States of America | Lumbar spinal surgery | Unclear | 14 | 13 | 27 | 61.0 | 58.0 | Anode: the superior motor cortex  Cathode: the DLPFC | Anode: the superior motor cortex  Cathode: the DLPFC | Maintain: 2 mA, 20 min | Maintain: 2 mA, 45 s |
| Dubois 2013 [26] | Belgium | Lumbar spinal surgery | General anesthesia | 40* | 19* | 63 | 50.3* | | Anode: the left DLPFC  Cathode: the scalp over the right ear | Anode: the left DLPFC  Cathode: the scalp over the right ear | Begin: a 8-s ramp­up phase  Maintain: 1 mA, 20 min  End: a 8­s ramp-down phase | Begin: a 8-s ramp­up phase + 1 mA, 14 s + a 8­s ramp-down phase  Maintain: none  End: none |
|  |  |  |  |  |  |  |  |  | Anode: the scalp over the right ear  Cathode: the left DLPFC |  |  |  |
| Borckardt 2013 [41] | United States of America | TKA | General anesthesia | 20 | 20 | 40 | 67* | | Anode: the motor strip  Cathode: the right DLPFC | Anode: the motor strip  Cathode: the right DLPFC | Begin: a ramp­up phase  Maintain: 2 mA, 20 min  End: a ramp-down phase | Begin: a ramp­up phase + 2 mA, 30 s + a ramp-down phase  Maintain: none  End: none |

**A**: active; **D**: day; **DLPFC**: dorsolateral prefrontal cortex; **M1**: primary motor cortex; **N**: no; **PCA**: patient-controlled analgesia; **PFC**: prefrontal cortex; **S**: sham; **S1**: primary somatosensory cortex; **tACS**: transcranial alternating current stimulation; **tDCS**: transcranial direct current stimulation; **THA**: total hip arthroplasty; **TKA**: total knee arthroplasty; **Y**: yes; -1D: 1 day before surgery; -0D: pre‑surgery on day of surgery; +0D: post‑surgery on day of surgery; +1D: postoperative day 1; +2D: postoperative day 2; +3D: postoperative day 3; +4D: postoperative day 4; +5D: postoperative day 5

*Sample size and age were reported for participants who completed the study, not the original randomized sample.

**Supplemental Table 3.** (Continued)

| **Study** | **Intervention** | | | | | **Included outcome** | **PCA (Y/N)** |
| --- | --- | --- | --- | --- | --- | --- | --- |
|  | **Intensity** | **Duration** | **Mode** | **Timing** | **Session count** |  |  |
| Antonenko 2026 [42] | 2 mA | 20 min | tACS | +0D | 1 | Postoperative delirium  Adverse events | N |
| Yang 2026 [37] | 2 mA | 19 min | tACS | +0D | 1 | Postoperative pain scores  Consumption of opioid drug  Quality of recovery  Adverse events | Y |
| Zan 2026 [38] | 2 mA | 20 min | tDCS | -1D, -0D, +1D, +2D, +3D, +4D, +5D | 7 | Postoperative pain scores  Postoperative delirium  Adverse events | Y |
| Wei 2026 [36] | 2mA | 20min | tDCS | -1D, +0D | 2 | Postoperative pain scores  Consumption of opioid drug  Postoperative delirium  Adverse events | Unclear |
| Yang 2025 [35] | 2 mA | 10 min | tDCS | +0D | 1 | Postoperative pain scores  Quality of recovery  Adverse events | Unclear |
| Yang 2025 [34] | 2 mA | 19 min | tDCS | -0D | 1 | Postoperative pain scores  Postoperative delirium  Adverse events | Y |
| Du 2025 [27] | 2 mA | 20 min | tACS | +0D | 1 | Postoperative pain scores  Consumption of opioid drug  Quality of recovery  Adverse events | Y |
| Li 2024 [10] | 2 mA | 20 min | tDCS | -1D, -0D | 2 | Postoperative pain scores  Postoperative delirium  Adverse events | Y |
| Tao 2024 [11] | 2 mA | 20 min | tDCS | +0D | 1 | Postoperative pain scores  Quality of recovery  Adverse events | Y |
| Yang 2024 [12] | 2 mA | 20 min | tDCS | +0D | 1 | Postoperative pain scores  Quality of recovery | Y |
| Tao 2023 [9] | 2 mA | 20 min | tDCS | +0D | 1 | Postoperative pain scores  Postoperative delirium  Adverse events | Y |
| Zhao 2022 [32] | 2 mA | 20 min | tDCS | During operation | 1 | Postoperative pain scores  Adverse events | Unclear |
| Hamed 2022 [28] | 2 mA | 20 min | tDCS | +0D, +1D, +2D | 3 | Postoperative pain scores  Consumption of opioid drug | Y |
| Stamenkovic 2020 [29] | 1.2 mA | 20 min | tDCS | +0D | 1 | Postoperative pain scores  Consumption of opioid drug  Adverse events | Y |
| Jiang 2018 [31] | 2 mA | 20min | tDCS | +1D | 1 | Postoperative pain scores  Adverse events | N |
| Ribeiro 2017 [33] | 2 mA | 20 min | tDCS | -1D, -0D | 2 | Postoperative pain scores | N |
| Khedr 2017 [30] | 2 mA | 20 min | tDCS | +0D, +1D, +2D, +3D | 4 | Postoperative pain scores  Consumption of opioid drug  Adverse events | N |
| Borckardt 2017 [39] | 2 mA | 20 min | tDCS | +0D 2 sessions, +1D 2 sessions | 4 | Consumption of opioid drug | Y |
| Glaser 2016 [40] | 2 mA | 20 min | tDCS | +0D 2 sessions, +1D 2 sessions | 4 | Consumption of opioid drug | Y |
| Dubois 2013 [26] | 1 mA | 20 min | tDCS | +0D | 1 | Postoperative pain scores  Consumption of opioid drug  Adverse events | Y |
| Borckardt 2013 [41] | 2 mA | 20 min | tDCS | +0D 2 sessions, +1D 2 sessions | 4 | Consumption of opioid drug | Y |

**A**: active; **D**: day; **DLPFC**: dorsolateral prefrontal cortex; **M1**: primary motor cortex; **N**: no; **PCA**: patient-controlled analgesia; **PFC**: prefrontal cortex; **S**: sham; **S1**: primary somatosensory cortex; **tACS**: transcranial alternating current stimulation; **tDCS**: transcranial direct current stimulation; **THA**: total hip arthroplasty; **TKA**: total knee arthroplasty; **Y**: yes; -1D: 1 day before surgery; -0D: pre‑surgery on day of surgery; +0D: post‑surgery on day of surgery; +1D: postoperative day 1; +2D: postoperative day 2; +3D: postoperative day 3; +4D: postoperative day 4; +5D: postoperative day 5

*Sample size and age were reported for participants who completed the study, not the original randomized sample.

**Supplemental Table 4.** Meta-analysis of outcomes at each time point after treatment.

| **Outcome** | **Follow-up time** | **Number of studies** | **Heterogeneity** | | **Results** | |
| --- | --- | --- | --- | --- | --- | --- |
|  |  |  | ***P*** | ***I²* (%)** | **SMD (95%CI)** | ***P*- value** |
| **Postoperative pain scores** | 0D | 7 | < 0.01 | 77 | -0.30(-0.67 to 0.07) | 0.12 |
|  | 2D | 10 | < 0.01 | 89 | -0.88(-1.32 to -0.44) | < 0.01 |
|  | 3D | 9 | < 0.01 | 87 | -0.75(-1.18 to -0.32) | < 0.01 |
|  | 4D | 4 | 0.46 | 0 | -0.23(-0.56 to 0.10) | 0.17 |
|  | 5D | 3 | 0.44 | 0 | -0.37(-0.78 to 0.04) | 0.07 |
|  | 6D | 1 | / | / | -0.72(-1.36 to -0.08) | 0.03 |
|  | 7D | 2 | 0.67 | 0 | -0.247 (-0.494 to 0.000) | 0.050 |
|  | 1M | 1 | / | / | -0.46 (-0.87 to -0.05) | 0.03 |
|  | 3M | 1 | / | / | Unavailable | < 0.05 |
| **Cumulative opioid consumption** | 1D | 8 | 0.98 | 0 | -0.10(-0.27 to 0.07) | 0.26 |
|  | 2D | 6 | 0.17 | 35 | -0.54(-0.80 to -0.29) | < 0.01 |
|  | 3D | 4 | < 0.01 | 80 | -0.48(-1.07 to 0.10) | 0.11 |
|  | 4D | 2 | 0.54 | 0 | -0.47(-0.91 to -0.03) | 0.04 |
|  | 5D | 2 | 0.62 | 0 | -0.49(-0.93 to -0.05) | 0.03 |
| **Quality of recovery** | 0D | 1 | / | / | 0.93(0.51 to 1.35) | < 0.01 |
|  | 3D | 2 | 0.95 | 0 | 0.29(0.03 to 0.54) | 0.03 |
|  | 1M | 1 | / | / | 0.56(0.15 to 0.96) | < 0.01 |
|  | 3M | 1 | / | / | 0.70(0.29 to 1.12) | < 0.01 |

**D**: day; **M**: month; **SMD**: standardized mean difference

**Supplemental Table 5.** GRADE certainty of evidence assessment

| **Certainty assessment** | | | | | | | **№ of patients** | | **Effect** | | **Certainty** | **Importance** |
| --- | --- | --- | --- | --- | --- | --- | --- | --- | --- | --- | --- | --- |
| **№ of studies** | **Study design** | **Risk of bias** | **Inconsistency** | **Indirectness** | **Imprecision** | **Other considerations** | **[Active-tES]** | **[Sham-tES]** | **Relative (95% CI)** | **Absolute (95% CI)** |  |  |
| **Pain score** | | | | | | | | | | | | |
| 17 | randomized trials | serious^a^ | serious^b^ | not serious | not serious | dose response gradient^c^ | 905 | 869 | - | SMD **0.57 SD lower** (0.84 lower to 0.3 lower) | ⨁⨁⨁◯ Moderate^a,b,c^ | CRITICAL |
| **Total opioid consumption** | | | | | | | | | | | | |
| 10 | randomized trials | serious^a^ | serious^d^ | not serious | not serious | none | 422 | 364 | - | SMD **0.47 SD lower** (0.74 lower to 0.2 lower) | ⨁⨁◯◯ Low^a,d^ | IMPORTANT |
| **Quality of recovery** | | | | | | | | | | | | |
| 4 | randomized trials | serious^a^ | serious^e^ | not serious | serious^f^ | none | 201 | 200 | - | SMD **0.44 SD higher** (0.18 lower to 1.05 higher) | ⨁◯◯◯ Very low^a,e,f^ | IMPORTANT |
| **Postoperative delirium** | | | | | | | | | | | | |
| 6 | randomized trials | serious^a^ | serious^g^ | serious^f^ | not serious | strong association^h^ | 43/511 (8.4%) | 87/463 (18.8%) | **RR 0.45** (0.25 to 0.79) | **90 fewer per 1,000** (from 123 fewer to 34 fewer) | ⨁⨁◯◯ Low^a,f,g,h^ | IMPORTANT |
| **Nausea or vomiting** | | | | | | | | | | | | |
| 12 | randomized trials | serious^a^ | not serious | not serious | serious^f^ | none | 154/688 (22.4%) | 190/694 (27.4%) | **RR 0.84** (0.75 to 0.95) | **44 fewer per 1,000** (from 68 fewer to 14 fewer) | ⨁⨁◯◯ Low^a,f^ | IMPORTANT |
| **Headache or dizziness** | | | | | | | | | | | | |
| 11 | randomized trials | serious^a^ | not serious | not serious | not serious | none | 51/617 (8.3%) | 46/621 (7.4%) | **RR 1.01** (0.70 to 1.47) | **1 more per 1,000** (from 22 fewer to 35 more) | ⨁⨁⨁◯ Moderate^a^ | IMPORTANT |
| **Discomfort at the site of electrical stimulation** | | | | | | | | | | | | |
| 10 | randomized trials | serious^a^ | not serious | not serious | not serious | publication bias strongly suspected strong association^i,j^ | 90/742 (12.1%) | 18/672 (2.7%) | **RR 3.10** (1.96 to 4.90) | **56 more per 1,000** (from 26 more to 104 more) | ⨁⨁⨁◯ Moderate^a,i,j^ | IMPORTANT |

**CI:** confidence interval; **RR:** risk ratio; **SMD:** standardized mean difference

#### Explanations

a. Most studies were judged to have some concerns.

b. *I²* = 87%

c. Meta-regression showed that the treatment effect was associated with the number of stimulation sessions.

d. *I²* = 68%

e. *I²* = 89%

f. During sensitivity analysis, the effect size changed.

g. *I²* = 55%

h. RR = 0.45

i. Egger’s test and funnel plot indicated the presence of publication bias.

j. RR = 3.10

**Supplemental Fig. 1.** (a) Funnel plot for postoperative quality of recovery on day 1 [11,12,35,37]. (b) Funnel plot for postoperative delirium [9,10,34,36,38,42].

**Supplemental Fig. 2.** Subgroup analyses and univariate meta‑regression for total opioid consumption [26–30,36,37,39–41].

**Supplemental Fig. 3.** (a) Baujat plot for postoperative total opioid consumption. (b) Graphical display of study heterogeneity (GOSH) plot for postoperative total opioid consumption. (c) GOSH plot after excluding the Hamed 2022 study [28]. (d) GOSH plot after excluding the Du 2025 study [27]. (e) GOSH plot after excluding both studies [26–30,36,37,39–41].

**Supplemental Fig. 4.** (a) Trial sequential analysis (TSA) plot for postoperative total opioid consumption [26–30,36,37,39–41]. (b) TSA plot for postoperative quality of recovery on day 1 [11,12,35,37]. (c) TSA plot for postoperative delirium [9,10,34,36,38,42].
